# COVID-19 post-vaccination in healthcare workers and vaccine effectiveness, Brazil, 2021

**DOI:** 10.1016/j.clinsp.2022.100109

**Published:** 2022-09-12

**Authors:** Caio Medeiros Fernandes, Shirley L. Dias, Maira C. Ferreira, Expedito J.A. Luna

**Affiliations:** Hospital das Clínicas da Faculdade de Medicina da Universidade de São Paulo (HCFMUSP), São Paulo, SP, Brazil

**Keywords:** COVID-19, Clinical conditions, Healthcare workers, Vaccine effectiveness, Vaccine impact

## Abstract

**Objects:**

This study aimed to describe COVID-19 cases in healthcare workers at a large tertiary hospital, after a vaccination campaign, to understand the individual characteristics, timeliness, symptomatology, and severity of the conditions.

**Methods:**

The COVID-19 reporting files from the hospital's healthcare workers and their records in the vaccine registry were analyzed, regarding vaccination status, symptoms, sociodemographic characteristics, comorbidities, and outcomes. Vaccination descriptive analysis was carried out and the impact and effectiveness of vaccination in relation to symptomatic infection and hospitalization were estimated.

**Results:**

In a total of 696 PCR-confirmed COVID-19 patients, vaccination coverage for the 1^st^ and 2^nd^ dose was 92.8% and 85.5%. Patients with complete doses had a mean interval of 96.8 days between vaccination and the onset of symptoms. Of the 664 participants with available clinical data, 165 had at least 1 comorbidity. During the study, 12 patients were hospitalized, 58.3% with a complete vaccination schedule. Three of this group died. The effectiveness of vaccination for symptomatic cases and hospitalization was 22.1% and 69.0%, respectively. The impact of vaccination on symptomatic cases and hospitalization was 81.4% and 89.7%, respectively.

**Discussion:**

The majority of COVID-19 cases in the study were classified as mild. The impact of vaccination for confirmed cases was significant, both in reducing the incidence of symptomatic cases and hospitalizations. The presence of comorbidities in approximately ¼ of the patients increased the risk of these individuals. The mean time interval between diagnosis and the 2^nd^ dose of vaccine was longer in the hospitalized group, reinforcing the protective decline over longer periods.

## Highlights

• Vaccination with any vaccine was effective in preventing confirmed COVID-19 cases.
• CoronaVac was effective in preventing COVID-19 related hospitalizations.
• Vaccination had a significant impact in reducing cases and hospitalizations.
• Predominance of mild cases, with respiratory symptoms, myalgia, and headache.
• Vaccine protection waned over time.

## Introduction

On March 20, 2020, community transmission of the disease caused by the new 2019’s Coronavirus (COVID-19) was recognized by the Brazilian Ministry of Health. Due to its high transmissibility, it quickly spread throughout the country, with a variety of clinical presentations, from asymptomatic to fatal cases, drawing the attention of health authorities.^1^ One of the responses to the pandemic was the fast development and production of vaccines against the new pathogen. It was estimated that around 60%‒70% of the population would need to be vaccinated in order to reach a herd immunity state,^2^ which would be capable of containing the progression of the pandemic. Vaccination had already been shown to be effective in different regions. In Los Angeles, USA, the unvaccinated population presented a risk of infection and hospitalization 4.9 and 29.2 times, respectively, greater than the vaccinated group.^3^ In Israel, where massive immunization of the population was implemented, a 90.5% effectiveness for disease prevention after 2 doses was observed, reinforcing the need for a vaccine approach.^4^

In January 2021, two vaccines received authorization for emergency use granted by the Brazilian national health regulatory agency (ANVISA). One of them, CoronaVac, developed by the pharmaceutical company Sinovac in partnership with the Butantan Institute, is relevant for this study, it was the first SARS-CoV-2 vaccine available in Brazil, and as such, it was widely used to vaccinate the priority groups. The purpose of the vaccination program was to prevent severe cases of the disease,^5^ reducing the demand for hospital beds and intensive care units. The vaccine schedule consisted of two doses. Previous phase II studies had shown greater seroconversion with the 28-day schedule between them.^6^ The phase III clinical trial of CoronaVac showed an efficacy of 50.7% against symptomatic disease and 100% against moderate and severe cases.^7^ It was also found that healthcare professionals had the highest COVID-19 incidence among the groups included in the clinical trials.^7^

However, after the implementation of the vaccination campaign, the occurrence of symptomatic and even severe cases continued to occur, which could be explained by the reduction of vaccine protection over time and the emergence of new variants. In a study carried out in Israel, where a vaccination campaign with the BNT162b2 vaccine promoted massive coverage of the population, an increase in the number of cases was observed as a function of the time elapsed since vaccination, suggesting a decrease in its effectiveness for all ages, which led the local government to implement a booster dose.^8^

The majority of cases of COVID-19 in vaccinated people are mildly symptomatic or asymptomatic, in addition to presenting lower transmissibility when compared to the unvaccinated population.^9^ A greater severity is related to advanced age and the presence of comorbidities,^10^ which was also observed in the scenario prior to vaccination. However, few studies were carried out in the Brazilian population to assess the profile of individuals who developed the disease after receiving the two-dose schedule, in terms of symptoms, evolution and exposure period.

Thus, at a time of effervescence and urgency in the production of knowledge about the long-term effectiveness of vaccination at a population level, new studies need to be carried out to measure the protection given to individuals, in order to assess the positive impact promoted by the vaccine campaign instituted in early 2021. This study, therefore, consisted of a descriptive epidemiological study that aimed to analyze the cases of COVID-19 among healthcare workers at the Hospital das Clínicas, the teaching hospital of the University of São Paulo Medical School (HC-FMUSP) after receiving none, one or two doses of the CoronaVac vaccine in the campaign carried out in January and February 2021. In this way, understanding the incidence, individual characteristics, timeliness, symptomatology, and severity of post-vaccination cases, is intended to contribute to a better understanding of the protective effects of the vaccine.

## Methods

### Selection of participants

This is a descriptive study of post-vaccination COVID-19 cases among an intensely exposed population group. Due to the ease in access to notification data and the inclusion among the priority groups for vaccination, the group chosen for this study was the healthcare workers of HC-FMUSP, who tested positive for COVID-19 after the vaccination campaign carried out in the months of January and February 2021 with the CoronaVac vaccine. The main source of data was the disease's mandatory notification system of the HC (SCAE). Additional data were obtained from the national mandatory reporting systems of the Ministry of Health (e-SUS Notifica and Sivep-Gripe). Data on vaccination were obtained from the State of São Paulo's vaccination recording system against COVID-19 (VaciVida). Participants were analyzed for: Vaccination status with one or two doses of CoronaVac (or other available vaccines) or not vaccinated, in the period defined for the study; PCR confirmed diagnosis of symptomatic COVID-19; Sociodemographic characterization; Associated symptoms and comorbidities; Outcomes (outpatient resolution, resolution in hospital or death).

According to the Brazilian Ministry of Health's COVID-19 Surveillance Guide, confirmed cases of the disease in vaccinated individuals are those who received the COVID-19 vaccine and subsequently had flu-like syndrome or severe acute respiratory syndrome with a detectable molecular biology test result for SARS-CoV-2 performed by the RT-PCR or RT-LAMP method or a reagent antigen for SARS-CoV-2 result by an immunochromatography method. In the present study, considering the HC's vaccination period established from January to February 2021, only cases confirmed by RT-PCR from the beginning of March to the end of August 2021 were included (protective vaccine period of up to 6 months as reported by the manufacturer).

Thus, participants were classified into three sub-groups: healthcare workers who were not vaccinated, those who received only one dose and those who received both doses of CoronaVac during the vaccination campaign. Employees who, despite not having participated in the initial vaccination campaign offered between January and February (due to age or personal refusal), received doses of other vaccines, or of CoronaVac itself, but within the period of this study (until August 2021), were also included.

For the effectiveness calculations, cases were considered vaccinated when the onset of symptoms occurred after 14 days of receiving the second dose of the vaccine (period determined for seroconversion). In this way, the authors considered as unvaccinated cases all the patients who were not vaccinated, who did not have a complete vaccination schedule, and who received the diagnosis with an interval of less than 14 days from the second dose. Data on the target population for vaccination was obtained from the Immunization Reference Center of Hospital das Clínicas (CRIE).

### Data analysis

After the selection of patients, their notification forms were retrieved and a database was organized in the Microsoft Excel® platform, from which descriptive statistical analyses were carried out, using the IBM SPSS Statistics® version 25, and OpenEpi. The characteristics of the studied groups were analyzed, evaluating variables that could or could not be associated with the development of the disease after vaccination. Among the variables possibly associated with post-immunization illness, age, the number of vaccine doses, the time interval between them, the developed symptoms and the presence of comorbidities were investigated.

In addition, based on data from the vaccination campaign of HC-FMUSP healthcare workers obtained at the institution's vaccine clinic (CRIE-HC-FMUSP), it was possible to evaluate the effectiveness of vaccination in this population, with regard to symptomatic infection and hospitalization. Effectiveness was calculated as in the formula: Ef = Inv - Iv/Inv, in which Inv stands for the cumulative incidence in non-vaccinated, and Iv is the cumulative incidence among vaccinated. It was also possible to calculate the impact obtained with vaccination, comparing the incidence of COVID-19 during the pre-vaccination period (March 2020 to February 2021) with the post-vaccination period (March to August 2021). The impact was calculated as the proportional reduction in cumulative incidence in the post-vaccination period. The 95% Confidence Intervals for proportions were calculated. The same analysis was carried out excluding the patients that had received other vaccines, in order to specifically elucidate CoronaVac's effectiveness and impact.

### Ethical aspects

The study was performed using freely accessible secondary data from mandatory reporting systems for confirmed COVID-19 cases and vaccination. The database generated for the present study does not contain data that allow the identification of patients. The study was submitted and approved by the Ethics Committee for the Analysis of Research Projects (CAPPesq), Plataforma Brasil CAAE # 55035921.0.0000.0068.

## Results

Considering the protective potential of vaccines in a period of 6 months and that a large portion of the healthcare workers of HC-FMUSP participated in the campaign during the months of January and February, the notification forms analyzed included all confirmed cases of COVID-19 with the onset of symptoms from March 1st to August 31^st^ of 2021. It is worth mentioning that among the evaluated patients, those considered unvaccinated or who received doses of one of the vaccines approved by ANVISA in the period after the vaccination campaign from January and February until the end of the study (August 2021) were also evaluated.

In view of the above, a total of 696 healthcare workers were selected, having been diagnosed with COVID-19 confirmed by PCR performed at one of the HC-FMUSP care facilities. This population, which includes different areas of health care, is characterized by a majority of female workers (66.7%), with a wide age distribution, and mostly white (74.3%). Additional descriptions of the sample are shown in Table 1.

**Table 1:** Description of the sample of HC-FMUSP healthcare workers who developed symptomatic infection with a positive PCR test for SARS-CoV2 from March to August 2021.

Descriptors	Frequency (n)	Proportion (%)
Sex		
Male	232	33.3
Female	464	66.7
Age group		
16‒29	168	24.1
30‒39	181	26.0
40‒49	173	24.9
50‒59	119	17.1
60‒78	55	7.9
Schooling		
Elementary School	58	8.3
High School	218	31.3
University education	295	42.4
Missing	125	18.0
Race/Color		
Yellow	12	1.7
White	517	74.3
Black	84	12.1
Missing	83	11.9
Total	696	100.0

Of the 696 patients, vaccine data on the 1^st^ and 2^nd^ dose were not available in just 1 and 3 subjects, respectively, therefore removed from further analysis. Vaccination coverage with the 1^st^ dose corresponded to 92.8% (n = 646) and with the 2^nd^ dose to 85.5% (n = 595). Those who were vaccinated after receiving the diagnosis of COVID-19 were included in the group of unvaccinated. The description of these data can be found in Table 2.

**Table 2:** Distribution of HC employees, prior to the diagnosis of COVID-19, according to vaccine status for SARS-CoV-2, São Paulo, 2021.

Vaccine	Frequency (n)	Proportion (%)
1^st^ dose		
Yes	646	92.8
No	49	7.1
Missing	1	0.1
Total	696	100.0
2^nd^ dose		
Yes	595	85.5
No	98	14,0
Missing	3	0.5
Total	696	100.0

Despite that the vaccination campaign carried out in January and February for the employees of the HC-FMUSP just offered CoronaVac, 8.2% of the COVID-19 cases (n = 53), either by choice, by participation in one of the COVID-19 vaccine clinical trials, or by age outside the initial coverage, were vaccinated before or after this period, having received doses of Astra-Zeneca's COVID-19 vaccine (n = 51; 7.89%) or another uninformed manufacturer (n = 2; 0.31%).

There was also evidence of a significant association between increasing age and vaccination coverage, as shown in Table 3, so that employees with more advanced age were those who had a higher vaccination coverage, while younger workers had a higher proportion of unvaccinated, especially in relation to the second dose. This result was already expected, because older patients were considered at greater risk and, therefore, were placed as a priority in the vaccination campaign, both inside and outside the HC's complex.

**Table 3:** Distribution of HC healthcare workers diagnosed with COVID-19 according to age group and vaccination status, São Paulo, 2021.

Age group	1^st^ dose		2^nd^ dose	
	Yes	No	Yes	No
16‒29	145 (86.8%)	22 (13.2%)	118 (70.7%)	49 (29.3%)
30‒39	167 (92.3%)	14 (7.7%)	160 (88.4%)	21 (11.6%)
40‒49	166 (96.0%)	7 (4.0%)	155 (90.6%)	16 (9.4%)
50‒59	114 (95.8%)	5 (4.2%)	111 (93.3%)	8 (6.7%)
60‒78	54 (98.2%)	1 (1.8%)	51 (92.7%)	4 (7.3%)
Total	646 (92.9%)	49 (7.1%)	595 (85.9%)	98 (14.1%)

Age range: 1^st^ dose ‒ Chi-Square of linear association p = 0.000

Age range: 2^nd^ dose ‒ Chi-Square of linear association p = 0.000

**Fig. 1:**
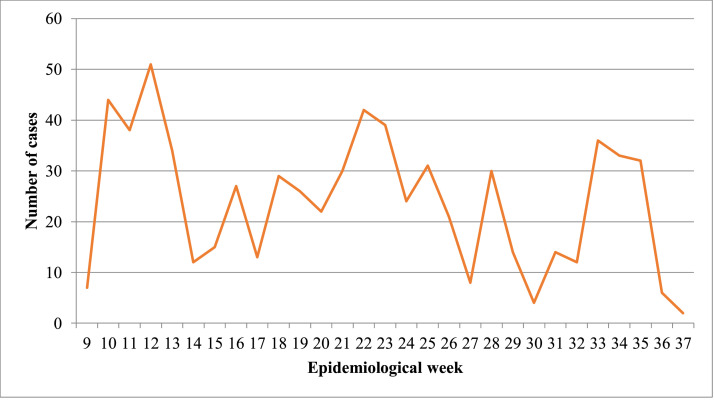
Distribution of confirmed cases of COVID-19 in HC healthcare workers according to epidemiological week of symptoms onset, from March to August 2021.

In order to compare the distribution of cases in the city of São Paulo, the epidemiological week of the cases’ onset of symptoms was analyzed. A larger concentration of cases was observed in the months of March, May and August, as shown in Fig. 1.

To determine the protective period provided against symptomatic SARS-CoV-2 infections, the time interval between the onset of symptoms and the 1^st^ and 2^nd^ dose of the 646 healthcare workers was analyzed, as shown in Table 4. For this analysis, 4 of them who had participated in previous clinical trials in 2020, having received doses in a period prior to the focus of this study, were excluded. In addition, the dates referring to the 1^st^ dose of 3 patients and the 2^nd^ dose of 1 patient were not registered in the medical records, nor at the VaciVida portal, and were also excluded from the analysis.

**Table 4:** Interval between the 1^st^ and 2^nd^ dose and the onset of symptoms in HC-FMUSP healthcare workers diagnosed with COVID-19 in the period from March to August 2021.

Range of days	1^st^ dose	2^nd^ dose
15 or less	24 (3.8%)	27 (4.6%)
16‒30	17 (2.7%)	65 (11.0%)
31‒60	105 (16.4%)	86 (14.6%)
61‒90	80 (12.5%)	94 (15.9%)
91‒120	100 (15.7%)	115 (19.5%)
121‒150	112 (17.5%)	78 (13.2%)
151‒180	78 (12.2%)	54 (9.2%)
181‒210	68 (10.6%)	67 (11.4%)
211‒240	51 (8.0%)	1 (0.17%)
241 or more	4 (0.6%)	3 (0.51%)
Total	**639 (100.0%)**	**590 (100.0%)**

1^st^ dose ‒ Mean: 118.1 / Standard deviation: 60.3. The 4 highest values, corresponding to employees vaccinated in clinical trials in 2020, were disregarded.

2^nd^ dose ‒ Mean: 96.8 / Standard deviation: 56.4 ‒ the 4 highest values, corresponding to employees vaccinated in clinical trials in 2020, were disregarded.

Thus, of the 646 vaccinated with the 1^st^ dose, 639 were validated and the mean interval for the onset of symptoms was 118.1 days (standard deviation 60.3), while of the 595 vaccinated with the 2^nd^ dose, the 590 analyzed had a mean interval of 96.8 days (standard deviation 56.4).

Regarding the clinical characteristics, of the 696 patients treated on an outpatient basis, data from 664 were available (the 32 missing patients did not have a completed form with the symptoms in the notification system). Only 1 of the registered patients (0.15%) had no symptoms, having been randomly tested in one of the outpatient clinics. The maximum number of symptoms recorded was 11, which was found in just 2 patients (0.3%). Most patients developed 4 to 6 symptoms (n = 394; 59.3%), followed by those who had 7 or more symptoms (n = 158; 23.8%) and those who had 1 to 3 symptoms (n = 111; 16.7 %). In the same analysis, of the 664 patients, the mean number of symptoms was 5.2 (standard deviation 1.7) in the 569 vaccinated with the 2^nd^ dose and 5.3 (standard deviation 2.1) in the ones not vaccinated with the 2^nd^, the difference was not significant (p = 0.537).

The quantitative description of the symptoms may be found in Table 5. The main symptoms, found in at least 50% of the evaluated patients, were coryza, cough, headache, myalgia, and sore throat.

**Table 5:** Clinical symptoms description of HC-FMUSP employees diagnosed with COVID-19, from March to August 2021.

Symptom	Frequency	Proportion
Coryza	526	79.2%
Cough	516	77.7%
Headache	490	73.8%
Myalgia	387	58.3%
Sore throat	358	53.9%
Adynamia (weakness)	289	43.5%
Olfactory disorders	222	33.4%
Fever	213	32.1%
Taste disorders	176	26.5%
Nausea/vomiting	89	12.8%
Diarrhea	79	11.9%
Others	77	11.6%
Difficulty breathing	69	10.4%
Abdominal pain	20	3.0%
Musculoskeletal pain	25	3.7%
Gynecological complaints	2	0.3%
Rash	1	0.2%
Irritability/confusion	1	0.2%
Emotional disorders	‒	0.0%

Regarding the statistical significance of the association between symptoms and exposure to the 2^nd^ dose of vaccine, diarrhea (p = 0.017), nausea/vomiting (p = 0.029), and adynamia (p = 0.001) were more present in the non-vaccinated group, while coryza (p = 0.001) and myalgia (p = 0.022) were more frequent in the group vaccinated with the 2^nd^ dose. Additional data are presented in Table 6.

**Table 6:** Association between exposure to the 2^nd^ dose of the vaccine and the symptoms developed by HC-FMUSP employees diagnosed with COVID-19, March to August 2021.

	2^nd^ dose of vaccine		
Symptoms	Yes	No	p-value
Coryza			0.001
Yes	464 (81.5%)	62 (66.7%)	
No	105 (18.5%)	31 (33.3%)	
Adynamia (weakness)			0.001
Yes	233 (40.9%)	55 (59.1%)	
No	336 (59.1%)	38 (40.9%)	
Diarrhea			0.017
Yes	61 (10.7%)	18 (19.4%)	
No	508 (89.3%)	75 (80.6%)	
Myalgia			0.022
Yes	341 (59.9%)	44 (47.3%)	
No	228 (40.1%)	49 (52.7%)	
Nausea/Vomiting			0.029
Yes	69 (12.1%)	19 (20.4%)	
No	500 (87.9%)	74 (79.6%)	
Cough			0.162
Yes	447 (78.6%)	67 (72.0%)	
No	122 (21.4%)	26 (28.0%)	
Abdominal pain			0.182^a^
Yes	15 (2.6%)	5 (5.4%)	
No	554 (97.4%)	88 (34.6%)	
Difficulty breathing			0.204
Yes	55 (9.7%)	13 (14.0%)	
No	514 (90.3%)	80 (86.0%)	
Musculoskeletal pain			0.378^a^
Yes	20 (3.5%)	5 (5.4%)	
No	548 (96.5%)	88 (94.6%)	
Headach			0.535
Yes	417 (73.3%)	71 (76.3%)	
No	152 (26.7%)	22 (23.7%)	
Fever			0.595
Yes	180 (31.6%)	32 (34.4%)	
No	389 (68.4%)	61 (65.6%)	
Sore throat			0.678
Yes	305 (53.6%)	52 (55.9%)	
No	264 (46.4%)	41 (44.1%)	
Taste disorder			0.688
Yes	152 (26.7%)	23 (24.7%)	
No	417 (73.3%)	70 (75.3%)	
Olfactory disorder			0.830
Yes	190 (33.4%)	30 (32.3%)	
No	379 (66.6%)	63 (67.7%)	
Irritability/ Confusion			1.000^a^
Yes	1 (0.2%)	0 (0.0%)	
No	568 (99.8%)	93 (100.0%)	
Rash			1.000^a^
Yes	1 (0.2%)	0 (0.0%)	
No	568 (99.8%)	93 (100.0%)	
Gynecological complains			1.000^a^
Yes	2 (0.4%)	0 (0.0%)	
No	567 (99.6%)	93 (100.0%)	
Emotional disorders			‒
Yes	0 (0.0%)	0 (0.0%)	
No	569 (100.0%)	93 (100.0%)	
Total	**569 (100.0%)**	**93 (100.0%)**	**‒**

Total records analyzed ‒ 662 (94.8%) / 36 missing (5.2%).

a Fisher's Exact Test.

The presence of comorbidities was also evaluated. Of the 664 participants with data on this variable, 499 patients (75.2%) did not have any comorbidity and 165 (24.8%) had at least 1 (decompensated chronic respiratory diseases, advanced stage chronic kidney diseases, chromosomal diseases, immune fragility status, chronic heart disease, diabetes, immunosuppression, pregnancy, postpartum women, and obesity). Of this group, 154 reported 1 or 2 comorbidities (23.2%) and 11 with 3 or 4 (1.6%). There was no significant difference (p = 0.501) between the mean number of symptoms presented by patients with comorbidities (5.30) and without comorbidities (5.19).

Of the 696 employees initially treated on an outpatient basis, 12 were hospitalized in the study period. Their mean age was 51.5 years (range 73‒35) and 66.7% (n = 8) and 58.3% (n = 7) received the 1^st^ and 2^nd^ dose of the vaccine, respectively. Only 4 of the hospitalized patients (33.3%) did not receive any vaccine dose. The mean interval between the date of the second dose and the onset of symptoms was 119 days, for the patients who received both doses of the vaccine and had the vaccination date available (n = 6).

The mean number of symptoms presented by the hospitalized group was 5.9 and the more frequent ones (present in at least 50%) were fever (n = 9), cough (n = 8) and dyspnea (n = 10). In the group of those vaccinated with both doses the mean number of symptoms was 5.7, while in those vaccinated with one or no dose the mean was 6.25 symptoms. All hospitalized patients had at least one comorbidity.

Of the hospitalized patients, 9 were discharged (75.0%) and 3 died (25.0%). All three had received the second dose of CoronaVac, 57, 95 and 193 days before the onset of symptoms. The mean length of stay of the patients who were discharged was 9.5 days, with 6 days for those who received both doses and 13 days for those who received one or no dose of the vaccine.

To calculate the effectiveness of vaccination in relation to symptomatic cases of COVID-19 and hospitalization, two analyses were conducted: the first one including all vaccinated cases, regardless of the vaccine they received, and the second one including only those who received the CoronaVac's vaccine. For this purpose, population data obtained from vaccination call lists and vaccination records were used, resulting in a total of 25,442 HC's employees (21,590 vaccinated and 3,852 unvaccinated). For the second analysis, the 54 cases that received at least one dose of another vaccine than CoronaVac were excluded, totaling 25,388 employees (21,542 vaccinated and 3,846 unvaccinated).

The effectiveness of the vaccination with any vaccine for symptomatic cases and hospitalization was respectively 21.9% (95% CI 6.0%‒35.5%) and 68.8% (95% CI -6.6%‒90.9%), and -6.3% (95% CI -17.2%‒24.3%) and 73.2% (95% CI 5.2%‒92.4%) in the CoronaVac analysis (Table 7).

**Table 7:** Effectiveness of vaccination with any vaccine and vaccination with CoronaVac for symptomatic PCR confirmed COVID-19 cases and hospitalizations.

	Vaccinated (2 doses of any vaccine)	Unvaccinated
**Population**	21,590	3,852
**Outpatient cases of COVID-19**	563	129
**Incidence**	2.6%	3.4%
**Effectiveness for COVID-19 cases**	21.9% (95% CI 6.0%‒35.5%)	
**Hospitalized COVID-19 cases**	7	4
**Incidence**	0.0324%	0.1038%
**Effectiveness for hospitalization**	68.8% (95% CI -6.6%‒90.9%)	
	**Vaccinated (2 doses of CoronaVac)**	**Unvaccinated**
**Population**	21,542	3,846
**Outpatient cases of COVID-19**	547	92
**Incidence**	2.54%	2.39%
**Effectiveness for COVID-19 cases**	-6.3% (95% CI -17.2%‒24.3%)	
**Hospitalized COVID-19 cases**	6	4
**Incidence**	0.0279%	0.1040%
**Effectiveness for hospitalization**	73.2% (95% IC 5.2%‒92.4%)	

Finally, to calculate the impact of vaccination with any vaccine and CoronaVac's vaccination, the number of reported cases among HC's healthcare workers in the period from March 2020 to February 2021 (period without vaccination) was used, resulting in a total of 3,751 outpatient and 106 hospitalized cases. The impact on the number of cases and hospitalization was, respectively, 81.4% (95% CI 79.9%‒82.9%) and 89.6% (95% CI 80.7%‒94.4%) for a vaccination with any vaccine, and 82.9% (95% CI 81.5%‒84.3%) and 90.6% (95% CI 82.0%‒95.1%) for a vaccination with CoronaVac (Table 8).

**Table 8:** Impact of vaccination with any vaccine and with CoronaVac on symptomatic PCR confirmed COVID-19 cases and hospitalization.

Timeline	March 2020 – February 2021	March 2021 – September 2021 Any vaccine (CoronaVac)
Number of cases	3,751	696 (639)
Incidence	14.7%	2.7% (2.52%)
Impact on incidence	81.4% (95% CI 79.9%‒82.9%)	
	82.9% (95% CI 81.5%‒84.3%)	
Hospitalized cases	106	11 (10)
Incidence	0.4166%	0.0432% (0.0394%)
Impact on hospitalization	89.6% (95% CI 80.7%‒94.4%)	
	90.6% (95% CI 82.0%‒95.1%)	

## Discussion

Regarding the effectiveness of vaccination with any vaccine for symptomatic cases, it was lower compared to other published studies,^11^ while in the separate analysis of CoronaVac no significant effect was observed, which may be related to the greater exposure of the healthcare workers to patients diagnosed and hospitalized with COVID-19 at the HC-FMUSP, resulting in a greater risk of infection and, consequently, of developing symptomatic disease. On the other hand, the effectiveness of CoronaVac for hospitalization was significant, confirming its effectiveness against severe disease, as already shown in previous studies.^12^^,^^13^ However, the analysis of the effectiveness of hospitalization both for a vaccination with any vaccine and with CoronaVac was influenced by the small number of patients hospitalized in the study period. Thus, it is important to emphasize that the approval for emergency use of the CoronaVac vaccine can be considered an effective measure since the main objective established was to reduce the number of hospitalized patients and deaths, which is compatible with the results presented in this study.

The impact of vaccination with any vaccine and with CoronaVac's vaccination was substantial, with a sharp drop in the incidence of symptomatic COVID-19 confirmed by PCR and in the total hospitalizations, which is a result consistent with the objective of the vaccine campaign at the beginning of the pandemic in reducing the number and severity of cases.

The participants of the present study were subject to greater exposure to the virus and, consequently, a greater risk of infection, once HC-FMUSP was the major reference hospital for severe cases in São Paulo's Metropolitan Area. The lower number of cases in patients of more advanced age may be related to the vaccination coverage with the two doses, as this group was previously allocated as a priority in the vaccination campaign, and a large proportion of them responded to the vaccination call. It should also be considered that part of the older professionals in the hospital was moved to remote activities, which may have reduced their exposure.

The epidemic curve of cases in this study has a similar pattern to that observed in the city of São Paulo in the same period,^14^ showing, however, additional sharper peaks in the final months of the analysis, such as August. The difference between these distributions may be associated with a reduction in the protection offered by the vaccine over time, with a progressive increase in the number of cases up to 90 days after the 2^nd^ dose. Such reduction was also evidenced in a previous study carried out in Israel, in which the serum levels of antibodies, used as a parameter for protective evaluation, decreased with temporal progression, promoting an increase in infection rates, regardless of the circulating variant.^15^

In terms of the frequency of symptoms, all the respiratory conditions (coryza, cough, sore throat) were the most prevalent, in addition to the presence of myalgia, adynamia, and headache. The symptoms evidenced in this analysis were compatible with other studies that clinically evaluated healthcare workers diagnosed with COVID-19, with fever, changes in smell and taste, cough and headache being frequently reported.^16^, ^17^, ^18^ The difference in symptoms between individuals vaccinated with the 2^nd^ dose and those who were not vaccinated may be interpreted by a possible greater containment of the viral infection in the respiratory system of patients with a complete regimen of doses, reducing the occurrence of systemic conditions, such as the gastrointestinal symptoms, for example. The presence of comorbidities in approximately ¼ of the evaluated patients also reinforces the view that such individuals are a group at greater risk of developing a symptomatic infection, regardless of vaccination status.^10^

Furthermore, the pattern of hospitalized patients also followed a similar logic to that presented in non-hospitalized cases. In this group, the mean time interval between diagnosis and the 2^nd^ dose of the vaccine was even longer, reinforcing the protective decline over longer periods,^15^ in addition to having at least one comorbidity,^10^ favoring the evolution to more severe disease. Despite this, the number of these patients was considerably smaller compared to the total sample, and it should also be noted that the hospitalization of the vaccinated patients was resolved in a shorter interval than that of the unvaccinated. Such findings reiterate the purpose of the vaccine campaign to reduce the number of severe cases, which required intensive therapeutic interventions, and deaths. The results are also compatible with recent studies that evaluated CoronaVac in other risk groups (e.g., the elderly and pregnant women), showing higher protective rates against severe disease and mortality.^19^^,^^20^

The present study had limitations capable of interfering with some of the results. Firstly, just the cases of healthcare workers from the HC-FMUSP complex who had their diagnosis confirmed within the HC-FMUSP own services were included. HC-FMUSP healthcare workers who had their diagnosis outside HC-FMUSP complex were not analyzed, which may have underestimated the number of cases. Numerical limitations, as mentioned earlier, especially regarding hospitalized patients, may also have interfered with the vaccine effectiveness and impact findings.

Another important limiting factor was the system used to retrieve the clinical data of the patients included in the study, since the publicly accessible information platform provides a limited list of symptoms and comorbidities, not covering the plurality of possible presentations that would be considered relevant to the case description. In addition, as this population was placed as a priority in the vaccination campaign in early 2021, the analyzed sample had a high vaccination coverage, so the comparison of the evaluated outcomes (symptoms, hospitalization and deaths) between those vaccinated and those not vaccinated was not established from groups with similar numbers, which could lead to false conclusions due to the low frequency of some outcomes, such as deaths, for example. A limitation that is worth mentioning is that the impact calculation compared different time intervals (March 2020 to February 2021 and March 2021 to August 2020), which could have interfered with the results.

## Conclusion

The present study is relevant to the current scenario, in which the mass vaccination policy has been adopted as a major control measure during the COVID-19 pandemic. The present study reaffirms the protective potential offered by the vaccination against severe cases and deaths. The authors also emphasize that, despite the massive reduction in pandemic progression after vaccination campaigns in Brazil and worldwide, the population, especially the most exposed groups, older people, and those with comorbidities, is still susceptible to the development of symptomatic cases and, more rarely, to hospitalization and death, which may be related to the decline in vaccine protection over time and the emergence of new variants. Thus, the authors reinforce the need to apply new booster doses and invest in innovative scientific research for the development of increasingly efficacious and effective vaccines and drugs to finally win the fight against the Coronavirus disease.

## Authors' contributions

The data were collected by Dias SL, Ferreira MC and Fernandes CM. The data analysis was performed by Fernandes CM and Luna EJA. The manuscript draft and its final version were written by Fernandes CM and Luna EJA. All authors have reviewed and approved the final version of the manuscript.

## Financing

The study did not require any additional funding.

## Conflicts of interest

The authors declare no conflict of interests. Every author had full access to all of the data, including statistical reports and tables, and they all take responsibility for the integrity of the data and the accuracy of the analysis.
